# Unilateral Pulmonary Artery Agenesis in an Adult: A Case Report and Review of Literature

**DOI:** 10.1155/crra/1565940

**Published:** 2026-03-02

**Authors:** Madhuranjan J., Arun Karthick R.

**Affiliations:** ^1^ Department of Radiology, Kauvery Hospital, Salem, Tamilnadu, India

**Keywords:** congenital pulmonary vascular anomaly, CT pulmonary angiogram, pulmonary hypertension, systemic collaterals, unilateral pulmonary artery agenesis

## Abstract

**Background:**

Unilateral pulmonary artery agenesis (UPAA) is a rare congenital anomaly that may remain undiagnosed until adulthood, when patients present with exertional dyspnoea, recurrent respiratory infections, hemoptysis, or pulmonary hypertension.

**Case Presentation:**

A 55‐year‐old female presented with exertional dyspnoea and hypoxemia. Clinical evaluation and echocardiography revealed pulmonary arterial hypertension, cor pulmonale with preserved left ventricular systolic function.

**Imaging Findings:**

Chest radiography demonstrated cardiomegaly with prominent but otherwise normal hilar shadows. Computed tomography pulmonary angiography revealed complete absence of the right pulmonary artery, with the right lung supplied by extensive systemic collaterals. Diffuse mosaic attenuation was noted, reflecting chronic hypoperfusion.

**Management:**

The patient was managed conservatively with oxygen therapy and pulmonary vasodilator therapy, including ambrisentan and tadalafil, resulting in clinical stabilization.

**Conclusion:**

Unilateral pulmonary artery agenesis is a rare but important cause of pulmonary hypertension and cor pulmonale in adults. Computed tomography pulmonary angiography plays a pivotal role in diagnosis by accurately delineating vascular anatomy and collateral circulation, enabling appropriate management and prevention of complications.

## 1. Introduction

Unilateral pulmonary artery agenesis (UPAA) is a rare congenital anomaly characterized by the absence or hypoplasia of one of the pulmonary arteries, resulting in the blood supply to the affected lung being maintained through systemic collaterals in most cases diagnosed. It is also known as unilateral absence of pulmonary artery (UAPA) or proximal interruption of the pulmonary artery. The reported prevalence of the condition is approximately one in 200,000 population [1].

Unilateral pulmonary artery agenesis has been studied extensively over the years. In 1958, Elder et al. presented early radiographic and cardiopulmonary studies describing unilateral pulmonary artery absence/hypoplasia [2]. Pool et al. expanded understanding of embryology and emphasized the importance of altered pulmonary blood flow in pulmonary hypertension [3]. Shakibi et al. identified pulmonary hypertension (PH) in a proportion of cases and discussed surgical repair considerations [4]. Ten Harkel et al. emphasized the need for follow‐up, reporting development of PH in a substantial proportion of patients over time [5]. Bockeria et al. reviewed a large series and reported right‐sided involvement as more common, with variants of collateral blood supply including coronary–bronchial communication [6]. Steiropoulos et al. described a range of chest radiographic findings in a case series [7].

Understanding the embryological origins and anatomical features of this condition is crucial for accurate diagnosis and appropriate management due to its association with other cardiac anomalies. We present a case of unilateral pulmonary artery agenesis diagnosed in adulthood with extensive systemic collateral supply and pulmonary hypertension.

## 2. Case Report

A 55‐year‐old female presented with complaints of dyspnoea on exertion persisting for 1 week. She previously had a few milder episodes of breathlessness which were treated symptomatically elsewhere. Upon arrival, the patient exhibited mild basal crepitations on auscultation. SpO2 was 70% on room air, which improved to 95% with 4 L/min of oxygen. Respiratory rate was 24/min, BP was 140/80 mmHg, and PR was 70/min.

**Figure 1:**
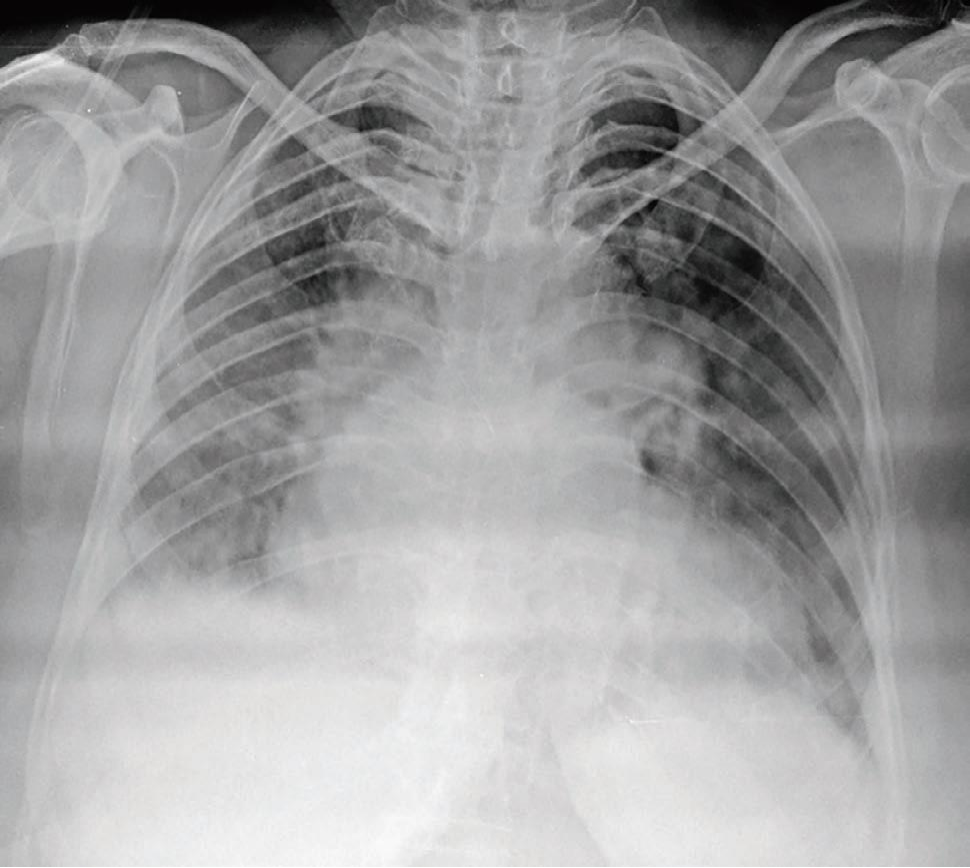
Chest radiograph (AP view) shows cardiomegaly, prominent hilar shadows, and diffuse haziness in lung fields. No significant loss of lung volume/mediastinal shift seen.

Chest radiograph (Figure 1) revealed cardiomegaly, prominent hilar shadows, and diffuse haziness in lung fields, consistent with pulmonary hypertension; no significant loss of lung volume or mediastinal shift was seen. The patient was initiated on oxygen support and admitted to the intensive care unit for stabilization. Transthoracic echocardiography revealed dilatation of the right atrium and right ventricle, a moderately elevated estimated pulmonary artery systolic pressure of approximately 42 mmHg, and preserved left ventricular systolic function with an ejection fraction of 60%. The patient was diagnosed with pulmonary hypertension and cor pulmonale and was started on diuretics.

**Figure 2:**
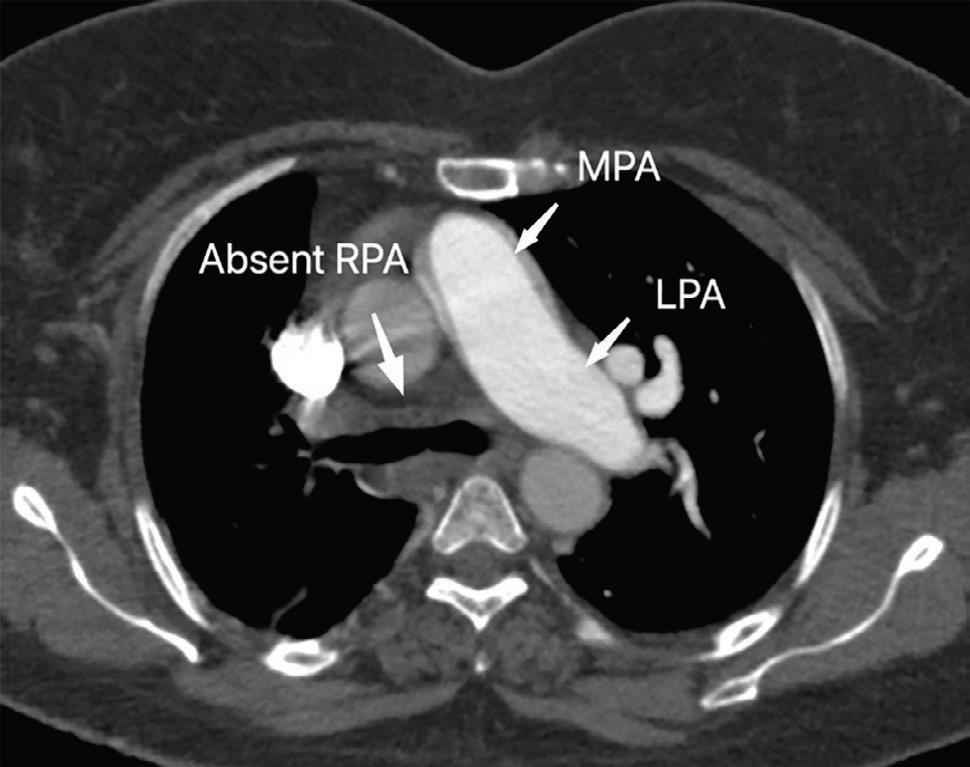
CT pulmonary angiogram phase in axial view shows complete absence of the right pulmonary artery (RPA). Main pulmonary artery (MPA) continues as left pulmonary artery (LPA).

Due to persistent hypoxia despite medical management and the diagnosis of pulmonary hypertension, computed tomography pulmonary angiography (CTPA) was performed in a Toshiba Aquilion 16‐slice multidetector CT scanner to rule out pulmonary embolism. Intravenous iodinated contrast (Iohexol 350 mgI/mL) was administered at a total volume of 90 mL, followed by imaging in both the arterial (pulmonary angiographic) and venous phases. Images were reconstructed and reviewed in the axial, coronal, and sagittal planes using multiplanar reconstruction techniques. It revealed the complete absence of the right pulmonary artery (Figure 2) and its branches, with the main pulmonary artery continuing as the left pulmonary artery. The right lung received blood supply from systemic collaterals, and subtle volume loss of the right lung was noted.

**Figure 3:**
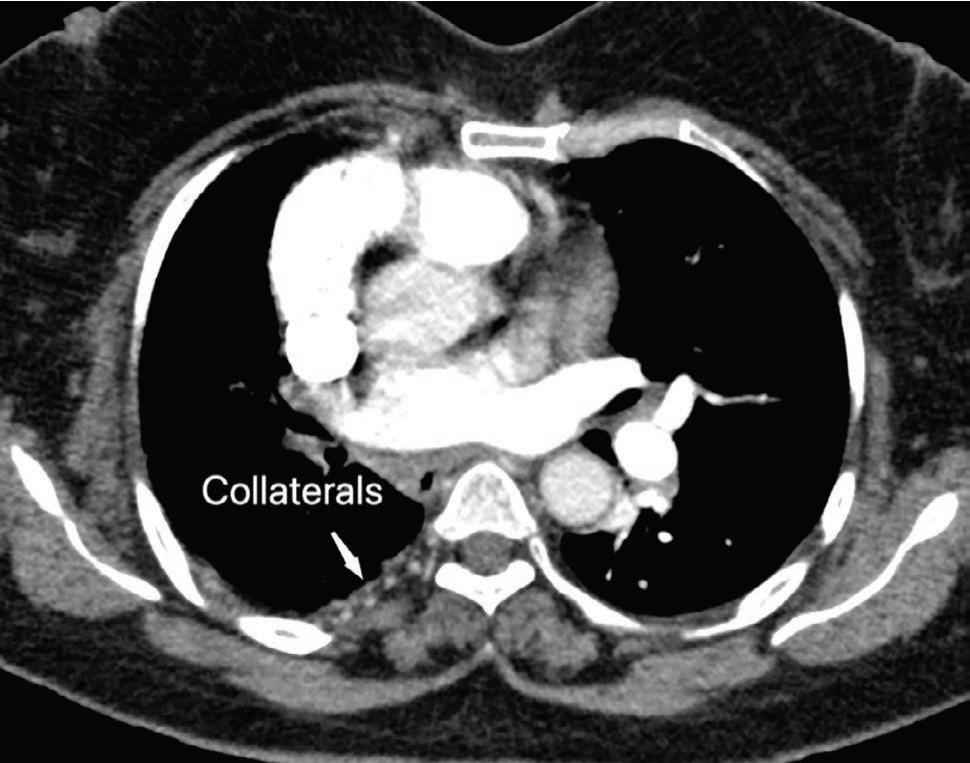
CT pulmonary angiogram phase in axial view shows tortuous, dilated right intercostobronchial arteries supplying the right lung (systemic collateral circulation).

**Figure 4:**
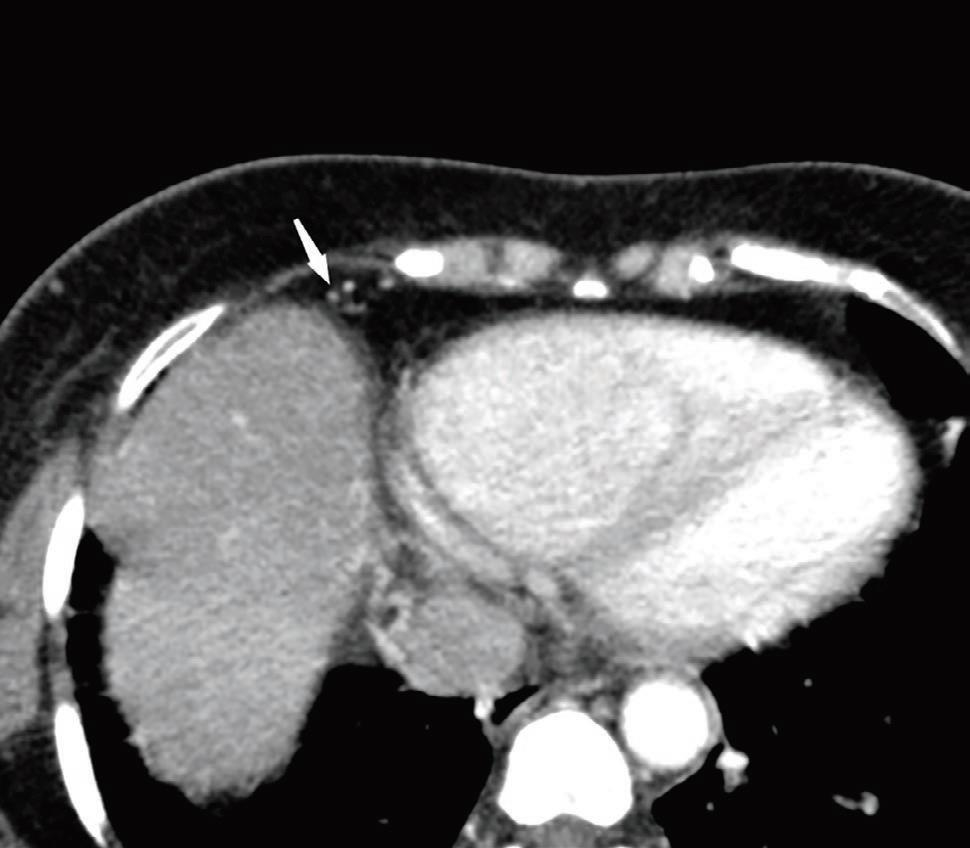
CT pulmonary angiogram phase in axial view shows collateral branch from right internal mammary artery supplying the right lung.

**Figure 5:**
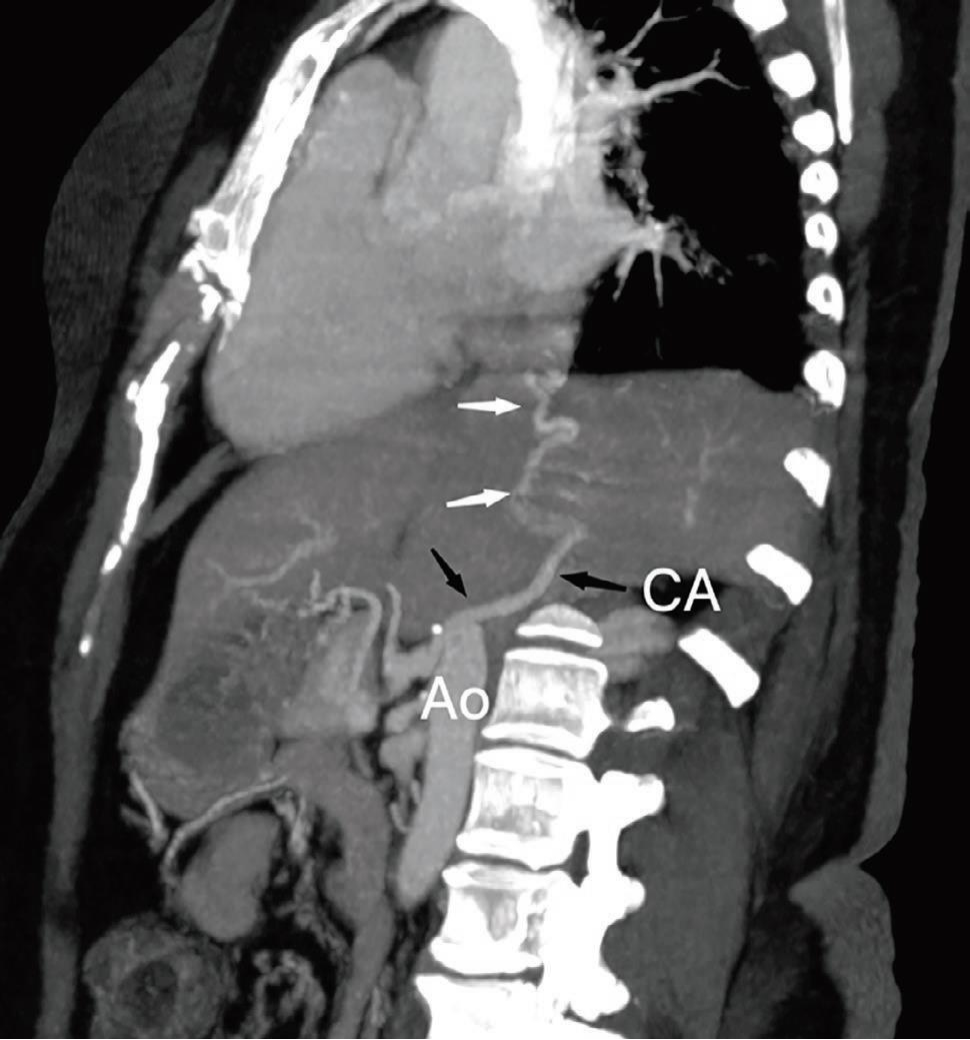
CT angiogram phase in curved multiplanar reconstruction and maximum intensity projection in sagittal view showing subdiaphragmatic collateral branch (white arrows) from celiac artery (CA) (black arrows) supplying the right lower lung.

**Figure 6:**
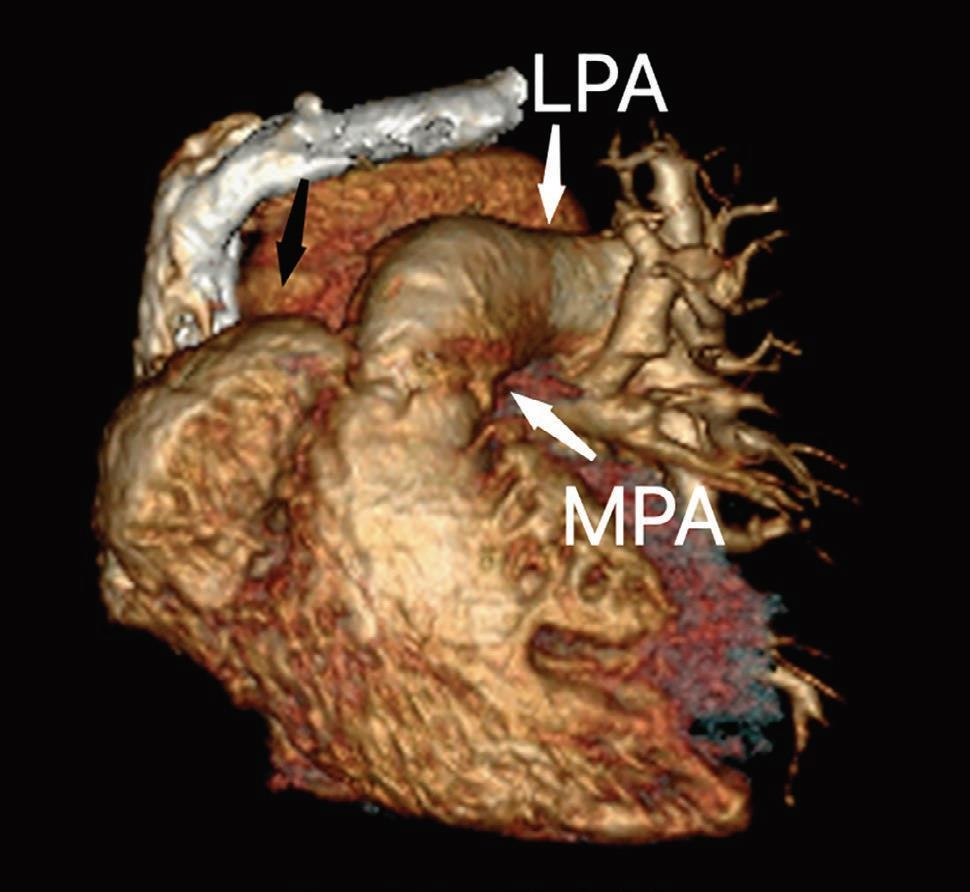
Volume‐rendered (VR) image in the anterior view showing absence of the right pulmonary artery in the expected position (black arrow), with the main pulmonary artery (MPA) continuing as the left pulmonary artery (LPA).

**Figure 7:**
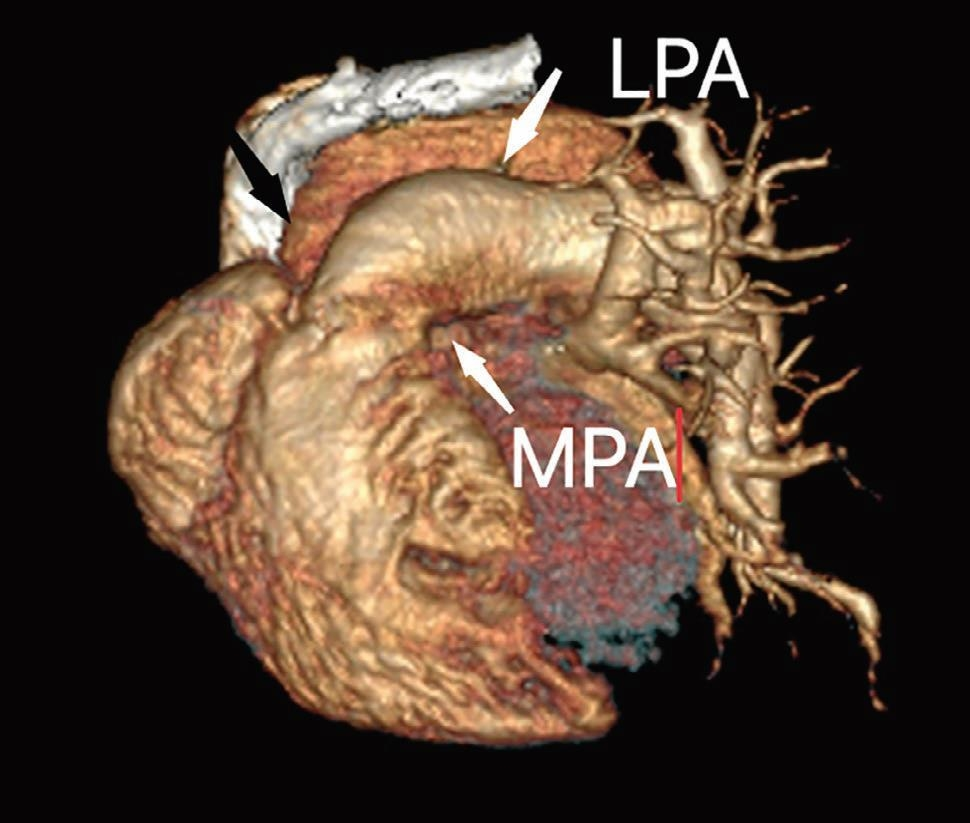
Volume rendered (VR) image in the oblique anterior view showing absent right pulmonary artery in the expected position (black arrow) with the main pulmonary artery (MPA) continuing as left pulmonary artery (LPA).

**Figure 8:**
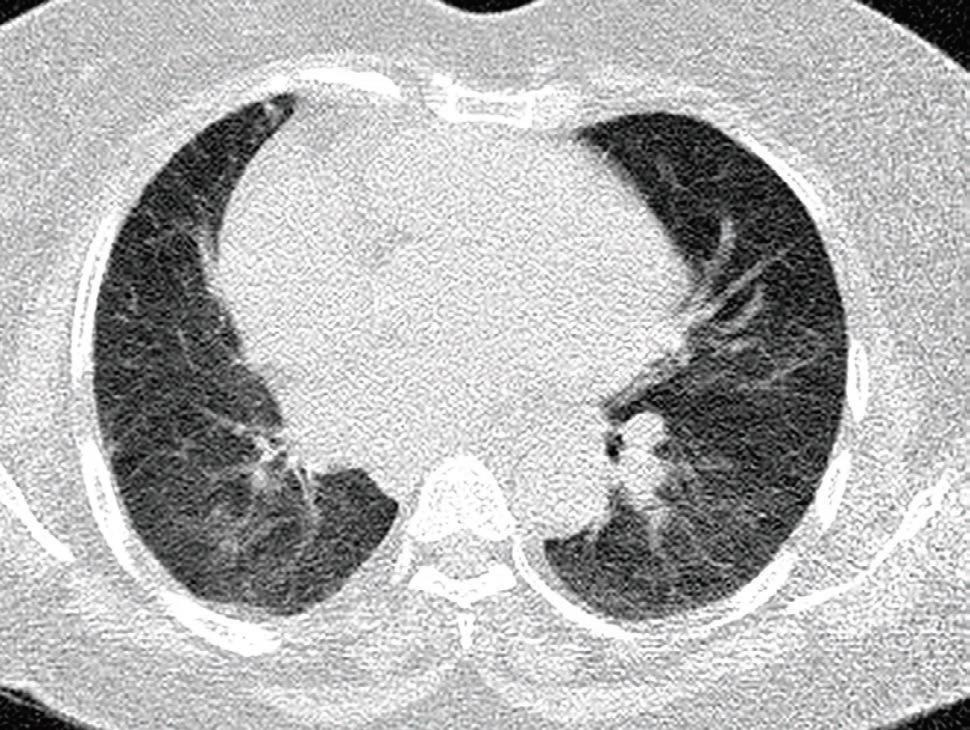
Axial CT images in lung window settings demonstrate mosaic attenuation (patchy regional differences in pulmonary attenuation) and cardiomegaly.

**Figure 9:**
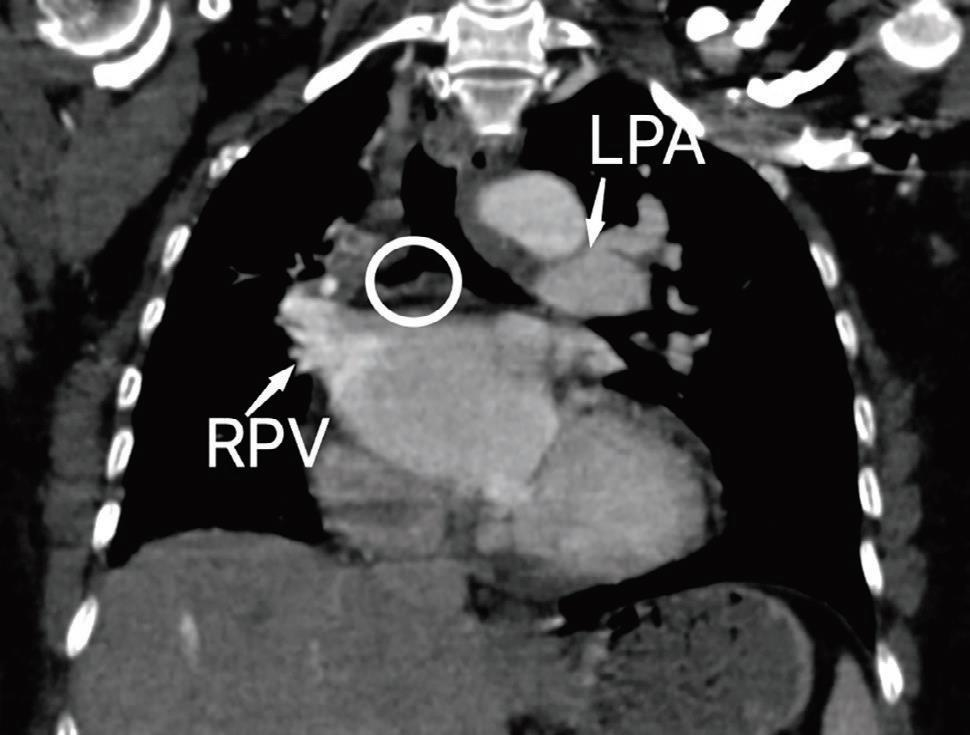
Coronal CT reconstruction image of CT chest in venous phase demonstrating the absence of right main pulmonary artery (white circle) and prominent right pulmonary vein (RPV). LPA—left pulmonary artery.

The right lung was supplied by prominent, tortuous systemic collaterals. Tortuous, dilated right intercostobronchial arteries were seen (Figure 3). A collateral branch from the right internal mammary artery was identified (Figure 4). A subdiaphragmatic collateral branch arising from the celiac artery supplied the right lower lung (Figure 5). Volume‐rendered images (VR = volume rendering) confirmed complete absence of the right pulmonary artery (Figures 6 and 7). Additional findings included cardiomegaly and diffuse mosaic attenuation (Figure 8). The reason for the apparently normal hilar shadows on the chest radiograph is unclear; however, this may be related to the prominence of the right pulmonary veins (Figure 9).

The main and left pulmonary arteries, including their branches, showed no evidence of pulmonary thromboembolism; however, mild dilatation of the main and left pulmonary arteries was noted, consistent with pulmonary arterial hypertension. No other associated cardiac anomalies were seen. The patient was managed medically in the intensive care unit using oxygen, ambrisentan, and tadalafil. The patient was stabilized and discharged with advice to avail oxygen at home.

## 3. Ethics Statement

Formal ethical approval was not required for this case report because the CT imaging was performed as part of routine clinical investigation, with no deviation from standard patient care and no additional procedures undertaken for research purposes. Informed consent was not obtained; however, all patient information and imaging data have been fully anonymised, and no identifiable details are included in this manuscript.

This case report was prepared in accordance with the CARE guidelines.

## 4. Discussion

### 4.1. Embryology and Pathophysiology

The origin of unilateral pulmonary artery agensis is attributed to the fetal period, where the proximal portion of the sixth aortic arch fails to develop, leading to the absence of the corresponding pulmonary artery [3]. In proximal interruption of unilateral pulmonary artery, there may be a short proximal pulmonary artery stump with atresia of the remaining artery. Despite the lack of a functional pulmonary artery, the affected lung receives blood supply through systemic collaterals, such as bronchial arteries, which can potentially lead to complications like hemoptysis [8]. Hemoptysis may be caused by hypertrophied systemic collateral branches from intercostal, bronchial, subclavian, and subdiaphragmatic arteries [9–11].

### 4.2. Associated Anomalies and Clinical Presentation

UPAA can be associated with cardiovascular anomalies (in about 60% of cases) such as septal defects (atrial septal defect or ventricular septal defect [13]), patent ductus arteriosus [13, 18], coarctation of the aorta, right aortic arch, truncus arteriosus, and tetralogy of Fallot [12]. As described by Bockeria et al., abnormal coronary–bronchial communications may also contribute to collateral blood flow to the affected lung [6]. Approximately 15% of patients are asymptomatic and may remain undiagnosed into adulthood [1]. Common symptoms include recurrent pulmonary infections, dyspnea, reduced exercise tolerance, pulmonary hypertension, and hemoptysis. Hemoptysis occurs in about 20% of patients [5].

### 4.3. Pulmonary Hypertension

Pulmonary hypertension can develop when blood flow is redirected from a missing pulmonary artery toward the contralateral functioning pulmonary artery, increasing flow and shear stress. This can promote release of vasoconstrictive mediators such as endothelin and, over time, lead to vascular remodelling, increased pulmonary vascular resistance, and pulmonary hypertension [14].

### 4.4. Imaging and Diagnostic Approach

Ventilation–perfusion scintigraphy demonstrates absent perfusion with preserved ventilation on the affected side [15]. Echocardiography is useful for identifying associated cardiac anomalies and estimating pulmonary pressures but may not directly visualize the absent pulmonary artery. Cardiac catheterization provides definitive diagnosis and hemodynamic data but is invasive and carries procedural risks [9–11]. CT has superior capability in delineating vascular anatomy and assessing collateral circulation.

Chest radiography may suggest the diagnosis when there is absence of a converging point of the pulmonary arteries at the hilum on the affected side [16]. Other features may include a hypoplastic lung with rib crowding, hemidiaphragm elevation, reduced pulmonary vascular markings, and mediastinal shift toward the affected side, with compensatory hyperinflation of the contralateral lung. However, radiographs may be nondiagnostic when pulmonary hypertension or congestion obscures typical signs, as in our patient.

CT/HRCT may show reduced lung volume and vascular markings on the affected side. Mosaic attenuation reflects regional differences in perfusion due to chronic underperfusion and ventilation–perfusion mismatch; relatively hypoperfused areas appear more lucent, whereas better‐perfused regions appear denser. Over time, recurrent infections and chronic hypoperfusion may contribute to bronchiectasis, interstitial thickening, fibrosis and occasional cystic changes; the contralateral lung may show compensatory overexpansion and increased vascularity [7, 17]. In our patient, the demonstration of extensive systemic collaterals (intercostobronchial, internal mammary, and celiac) supported the diagnosis and explained the risk profile for hemoptysis and pulmonary hypertension.

Although catheter pulmonary angiography has historically been regarded as the gold standard for evaluation of pulmonary arterial anatomy, it is an invasive procedure associated with procedural risks. In the present case, CT pulmonary angiography provided comprehensive, high‐resolution anatomical detail demonstrating the congenital absence of the pulmonary artery from its origin, associated lung volume loss, and systemic collateral supply. These findings were diagnostic and left no ambiguity regarding the underlying pathology.

Given the clarity of the diagnosis on CT and the absence of any anticipated therapeutic intervention that would require catheter‐based assessment, invasive pulmonary angiography was not considered necessary. CT pulmonary angiography was therefore deemed adequate for both diagnosis and clinical management.

Similarly, perfusion scintigraphy was not performed, as CT imaging had already established the diagnosis with high confidence, and additional functional assessment was unlikely to alter management or provide incremental diagnostic value.

Pulmonary artery agenesis should be differentiated from chronic thromboembolic occlusion, pulmonary artery hypoplasia, and postsurgical loss. Agenesis is characterized by congenital absence of the pulmonary artery from its origin, without a proximal stump, often with systemic collateral formation. Chronic thromboembolic disease typically shows residual arterial stumps or intraluminal thrombus, whereas postsurgical loss is suggested by prior intervention and operative changes. Hypoplastic pulmonary arteries are present but significantly reduced in caliber, maintaining continuity with the pulmonary trunk.

### 4.5. Management

Treatment approaches range from conservative management in asymptomatic cases to surgical and endovascular interventions in severe presentations. The overall mortality rate was reported as 7% [5], highlighting the importance of early detection and appropriate management. Pharmacological management of pulmonary hypertension using vasodilators and long‐term oxygen therapy for chronic hypoxemia is preferred [14]. Pneumonectomy or lobectomy was performed in 8% of patients, typically for recurrent hemoptysis or intractable infections. Revascularization of hidden pulmonary arteries was attempted in 7% of cases to improve pulmonary blood flow and alleviate symptoms [5]. Embolization of systemic‐to‐pulmonary collaterals is considered for massive hemoptysis.

### 4.6. What Makes This Case Noteworthy and Comparison With Major Series

This case is notable for late presentation (55 years) with hypoxemia and pulmonary hypertension, and for extensive systemic collateral supply including a subdiaphragmatic collateral arising from the celiac artery. In the large review by Bockeria et al., right‐sided UPAA was more common, consistent with our patient, and coronary–bronchial communications were described as an additional collateral pathway [6]. Ten Harkel et al. emphasized the importance of follow‐up, reporting pulmonary hypertension development in 44% of patients over time [5], which aligns with our patient′s presentation. The combination of adult diagnosis, pulmonary hypertension, and prominent systemic collaterals underscores the importance of CT angiography and angiographic confirmation in adult patients with unexplained pulmonary hypertension or hypoxemia.

## 5. Conclusion

Unilateral pulmonary artery agenesis is a rare cause of recurrent respiratory infections, hemoptysis, or pulmonary hypertension, and only a minority of patients remain asymptomatic into adulthood. Diagnosis can be established through chest radiography and contrast‐enhanced CT, whereas catheter pulmonary angiography remains the gold standard despite its invasive nature. In cases where large collateral arteries lead to pulmonary hypertension or hemoptysis, embolization techniques can be employed. Early diagnosis can prevent deterioration and mitigate potentially severe effects related to heavy exertion, sudden high‐altitude exposure, or pregnancy in an undiagnosed individual.

## Funding

No funding was received for this manuscript.

## Conflicts of Interest

The authors declare no conflicts of interest.

## Data Availability

Data sharing is not applicable to this article as no datasets were generated or analyzed during the current study.
